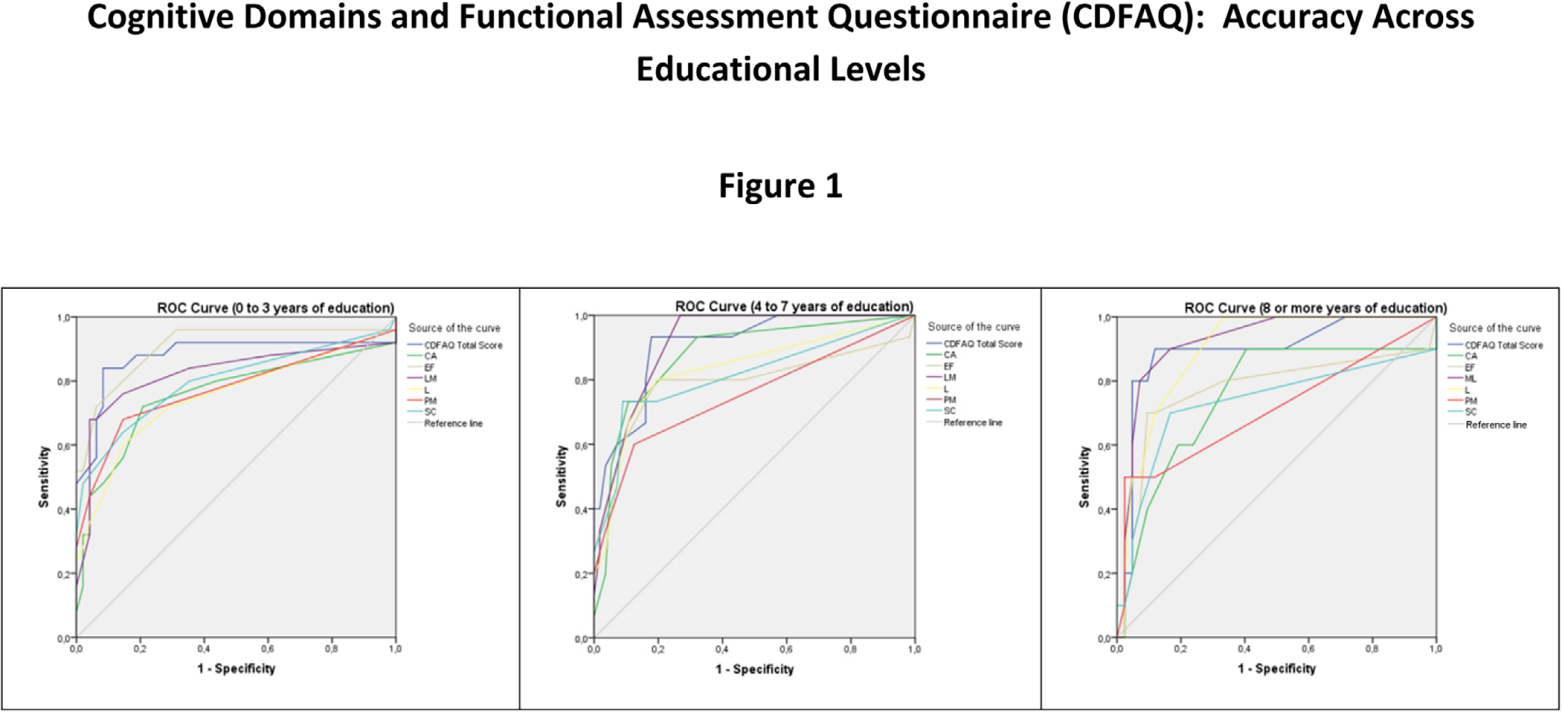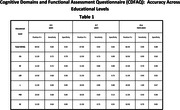# Cognitive Domains and Functional Assessment Questionnaire (CDFAQ): Accuracy Across Educational Levels

**DOI:** 10.1002/alz.092259

**Published:** 2025-01-03

**Authors:** Isabelle de Aguiar Maia, Caio Peixoto Tavares, Giovanna Correia Pereira Moro, Aline Siqueira de Souza, Jéssica Diniz Ferreira, Ivonne Carolina Bolaños Burgos, Gabriela Tomé Oliveira Engelmann, Maissa Ferreira Diniz, Marco Aurélio Romano‐Silva, Jonas Jardim de Paula, Maria Aparecida Camargos Bicalho, Bernardo de Mattos Viana

**Affiliations:** ^1^ Universidade Federal de Minas Gerais, Belo Horizonte, Minas Gerais Brazil; ^2^ Older Adult’s Psychiatry and Psychology Extension Program I Federal University of Minas Gerais, Belo Horizonte, Minas Gerais Brazil; ^3^ Older Adult’s Psychiatry and Psychology Extension Program (PROEPSI), School of Medicine, Universidade Federal de Minas Gerais (UFMG), Belo Horizonte, Minas Gerais Brazil; ^4^ Cog‐Aging Research Group, Universidade Federal de Minas Gerais (UFMG), Belo Horizonte, Minas Gerais Brazil; ^5^ Sciences Applied to Adult Health Postgraduate Program, School of Medicine, Universidade Federal de Minas Gerais (UFMG), Belo Horizonte, Minas Gerais Brazil; ^6^ Cog‐Aging Research Group, Belo Horizonte, Minas Gerais Brazil; ^7^ INCT – NeuroTecR and CTMM, Belo Horizonte, Minas Gerais Brazil; ^8^ Jenny de Andrade Faria Institute – Outpatient Reference Center for the Elderly, Universidade Federal de Minas Gerais (UFMG), Belo Horizonte, Minas Gerais Brazil; ^9^ Molecular Medicine Postgraduate Program, School of Medicine, Universidade Federal de Minas Gerais (UFMG), Belo Horizonte, Minas Gerais Brazil; ^10^ Department of Psychiatry, School of Medicine, Federal University of Minas Gerais, Belo Horizonte, Minas Gerais Brazil; ^11^ Molecular Medicine Postgraduate Program, Faculty of Medicine, Universidade Federal de Minas Gerais (UFMG, Belo Horizonte, Minas Gerais Brazil; ^12^ National Institute of Science and Technology Neurotec R (INCT‐MM), Belo Horizonte, Minas Gerais Brazil; ^13^ Molecular Medicine Program, Faculdade de Medicina, Belo Horizonte, Minas Gerais Brazil; ^14^ Department of Clinical Medicine, Faculdade de Medicina, Universidade Federal de Minas Gerais, Belo Horizonte, Minas Gerais Brazil

## Abstract

**Background:**

The Cognitive Domains and Functional Assessment Questionnaire (CDFAQ) assess cognitive and functional decline for Neurocognitive Disorders based on the DSM‐5 criteria (1). It’s accuracy to the Informant Questionnaire on Cognitive Decline in the Elderly ‐ Long Version (IQCODE‐LV) has been assessed (2), and was translated and validated into English. The informant version (CDFAQ‐IV) assess: Complex Attention (CA), Executive Functions (EF), Learning and Memory (LM), Language (L), Perceptual‐Motor (PM) and Social Cognition.

**Methods:**

To evaluate the CDFAQ‐IV’s accuracy to IQCODE‐LV across participant’s educational levels. Both questionnaires were applied to 196 informants of older adults. Older adults were stratified by educational levels into three groups: 73 participants had low educational level (0‐3 years), 71 had middle educational level (4‐7 years), and 52 with high educational level (8 or more years). Accuracy assessment was made by Receiver Operating Characteristic (ROC) and cut‐off values defined by Youden’s J statistic. This study was approved by the ethics committee of UFMG.

**Results:**

The low educational group had good accuracy (Area Under the Curve = 0.879) for the CDFAQ‐IV total score, excellent accuracy for the EF domain (AUC = 0.907), good accuracy for LM domain (AUC = 0.822) and poor accuracies for other domains. For the middle educational group, CDFAQ‐IV (AUC = 0.908) and LM domain (AUC = 0.914) showed excellent accuracies, while CA (AUC = 0.876), L (AUC = 0.832) and SC (AUC = 0.814) domains showed good accuracies. The others domains showed poor accuracies. For high educational group, LM domain (0.924) had excellent accuracy, while CDFAQ’s total score (AUC = 0.894) and L domain (AUC = 0.898) showed good accuracy. Poor accuracies were found for the other domains.

**Conclusions:**

CDFAQ’s total score shows good accuracy in samples of low and high educational levels and excellent accuracy in samples with 4 to 7 years of schooling. Interestingly, the LM domain shows excellent accuracy (AUC > 0.9) in samples with higher levels of education. Brazilian older adults have lower educational levels and more heterogeneity in schooling quality compared to those from high‐income nations. Assessment of psychometric properties according to education is needed, as well as more scenarios such as studies in community and clinical settings.